# Virtual Reality–Based Social Musical Exergame Guided by Self-Determination Theory for Young Adults With Depression and Anxiety: Protocol for a Randomized Controlled Trial

**DOI:** 10.2196/83737

**Published:** 2026-05-01

**Authors:** GuangZheng Wan, Yu Zhang, MengQi Li

**Affiliations:** 1 School of Medicine Hunan Traditional Chinese Medicine College Zhuzhou, Hunan China; 2 Chengdu University Chengdu China; 3 Changsha Special Education School Changsha China

**Keywords:** virtual reality, exergaming, depression, anxiety, young adults, randomized controlled trial, self-determination theory, digital mental health

## Abstract

**Background:**

Depression and anxiety frequently emerge during late adolescence and young adulthood; however, many conventional and app-based interventions struggle to sustain engagement. Virtual reality (VR) exergaming, music-based activities, and social interaction each show promise for supporting young people’s mental health, but their combined therapeutic value remains insufficiently tested.

**Objective:**

This study aims to evaluate the effectiveness of a 6-week VR-based social musical exergame for reducing depressive and anxiety symptoms in young adults. The secondary objectives are to explore whether changes in basic psychological need satisfaction are associated with symptom change and to assess the effects on loneliness, presence, cardiorespiratory fitness, and in-game music-movement synchronization.

**Methods:**

This study is a 3-arm, parallel-group randomized controlled trial. A total of 110 participants aged 18 to 25 years with mild to moderate depression or anxiety will be recruited and randomized in a 1:1:1 ratio to (1) a VR social musical exergame, (2) a matched VR solo musical exergame active control, or (3) a waitlist control receiving standardized mental health guidance. Assessments will be completed at baseline, at the postintervention assessment (week 6), and at the 1-month follow-up assessment (week 10). The primary planned comparison is the experimental group vs the active control group.

**Results:**

This study was approved by the ethics committee of Hunan Traditional Chinese Medical College on September 8, 2025 (YXLL202509006) and prospectively registered at ClinicalTrials.gov on March 15, 2026 (NCT07482852). Internal institutional funding had been secured. As of April 2026, the trial status was “not yet recruiting;” no participants had been enrolled, and no data analysis had been conducted. Recruitment is anticipated to begin in May 2026, with primary completion on March 1, 2028, study completion on May 1, 2028, and publication of the primary findings expected in late 2028.

**Conclusions:**

This protocol describes a self-determination theory–informed, multicomponent VR intervention designed to evaluate whether adding a bundled social layer to a matched solo exergame improves short-term mental health outcomes. The trial is expected to provide initial evidence on efficacy, safety, and potential mechanisms while generating hypotheses for future dismantling and longer-term trials.

**Trial Registration:**

ClinicalTrials.gov NCT07482852; https://clinicaltrials.gov/ct2/show/NCT07482852

**International Registered Report Identifier (IRRID):**

PRR1-10.2196/83737

## Introduction

### Background and Rationale

Late adolescence and young adulthood (ages 18-25 years) represent a high-risk window for the onset of mental health disorders, with a large proportion of lifetime conditions first emerging by the mid-20s [1-4]. Depression and anxiety in this developmental period are shaped by interacting social, psychological, and biological processes and remain a substantial public health concern [5-7].

However, conventional interventions such as counseling and health education often struggle to sustain participation, and this limitation extends to many digital mental health tools. Even when mental health apps are widely available, long-term engagement remains low, suggesting that motivational design is a persistent implementation challenge [8-10].

The convergence of immersive technologies and psychologically informed intervention design offers a promising alternative. Virtual reality (VR) can deliver embodied and engaging exergaming experiences [11-16], music can support emotional regulation and social bonding [17-19], and socially shared or synchronized activity may reduce loneliness and enhance connectedness [20-28]. Although VR-based mental health interventions have shown encouraging results for distress reduction [22,29-32], these components have often been studied in isolation. To address this gap, this trial is informed by self-determination theory (SDT), which proposes that autonomy, competence, and relatedness are basic psychological needs linked to intrinsic motivation and well-being [33,34]. Importantly, this intervention is designed as a bundled SDT-informed intervention rather than an experimental test of each need in isolation. The study therefore evaluates the effectiveness of a multicomponent social VR musical exergame and explores whether changes in need satisfaction are associated with symptom change; these exploratory analyses are hypothesis generating and do not permit causal conclusions about individual SDT mechanisms.

### Objectives

#### Primary Objective

The primary objective of this study is to determine the effectiveness of a 6-week VR social musical exergame intervention in reducing symptoms of depression and anxiety in young adults, compared with a matched active control group and a waitlist control group.

#### Secondary Objectives

The secondary objectives of this study are as follows:

- To explore whether changes in basic psychological need satisfaction are associated with changes in depression and anxiety symptoms. These analyses are specified as exploratory and hypothesis generating rather than as definitive tests of temporal mediation.
- To explore whether in-game performance (ie, music-movement synchronization) moderates competence satisfaction and mental health outcomes as an exploratory secondary analysis.
- To assess the intervention’s effects on loneliness, sense of presence, and cardiorespiratory fitness.

## Methods

### Study Design

This study will use a 3-arm, parallel-group, single-center randomized controlled trial design. Assessments will be conducted at baseline (T0), immediately after the intervention (T1, week 6), and at a 1-month follow-up (T2, week 10) to evaluate short-term maintenance of effects. The primary planned comparison is the experimental group vs the active control group, which isolates the added value of the social layer over a matched solo VR intervention. Comparisons involving the waitlist control are secondary. This protocol was prepared in accordance with the SPIRIT (Standard Protocol Items: Recommendations for Interventional Trials) guidelines.

### Study Setting

Participants will be recruited from local universities, vocational colleges, and community health centers in Changsha, China.

### Recruitment

Recruitment strategies will include posters and flyers at universities, vocational colleges, and community centers; online announcements; and referrals from collaborating educational and community services.

### Participant Timeline

Following screening and informed consent, eligible participants will complete baseline assessment (T0), undergo randomization, receive the 6-week intervention or waitlist condition, complete the postintervention assessment at week 6 (T1), and complete the follow-up assessment at week 10 (T2; Table 1).

**Table 1:** Schedule of enrollment, interventions, and assessments.

Time point	Enrollment	Allocation	T0^a^	T1^b^	T2^c^
Eligibility screen	✓				
Informed consent	✓				
Randomization		✓			
Intervention			✓		

^a^T0=baseline: week 0.

^b^T1=posttest: week 6.

^c^T2=follow-up: week 10.

### Participants

A total of 110 young adults aged 18 to 25 years will be recruited. The eligibility range was narrowed to 18 to 25 years to improve developmental homogeneity and align the sample with late adolescence and emerging adulthood, a period marked by elevated risk for depression and anxiety [1-4]. The participant eligibility criteria are shown in Textbox 1. Participants will be recruited through advertisements at local universities, vocational colleges, and community health centers.

**Participant eligibility criteria.**

**Inclusion criteria**

- Aged 18 to 25 years
- Meeting criteria for mild to moderate depression (Beck Depression Inventory-II score: 14-28) or anxiety (Beck Anxiety Inventory score: 8-25)

**Exclusion criteria**

- A diagnosis of a severe mental illness (eg, psychosis) or an acute psychiatric crisis requiring immediate treatment
- Significant virtual reality (VR) motion sickness or other contraindications to safe VR participation
- Current engagement in other forms of systematic psychotherapy

### Sample Size Calculation

An a priori power analysis was conducted using G*Power. On the basis of meta-analyses of related VR-based mental health interventions [30,31], a medium effect size (f=0.25) was assumed. To achieve 80% power at an alpha level of .05 for the primary group-by-time comparison, a required sample of 90 participants was indicated. To account for an anticipated attrition rate of 20% to 30%, consistent with rates reported in VR intervention literature, the recruitment target is set at 110 participants. A sensitivity analysis indicates that with 30% attrition (leaving approximately 77 participants), statistical power for the primary comparison would be reduced to approximately 70%, which is acknowledged as a limitation. To minimize attrition, the study will implement flexible scheduling, session reminders, brief mid-study check-ins, and graduated VR exposure in the first session to identify and manage cybersickness early. If observed attrition exceeds 30%, this will be reported transparently and considered in the interpretation of results. This sample size determination was made for the primary clinical outcomes and was not specifically powered for mediation or moderation analyses; those analyses will therefore be treated as exploratory.

### Interventions

#### Experimental Group: VR Social Musical Exergame

Participants in this arm will engage in the VR intervention twice a week for 45 minutes over 6 weeks. Prior to the first intervention session, all participants in VR conditions (experimental group and active control group) will complete a 15-minute familiarization session using a neutral, nontherapeutic VR environment to reduce initial novelty effects and identify individuals who may experience significant cybersickness. Waitlist control participants will receive a brief (5-minute) VR demonstration to partially equalize technology exposure. The experimental condition is conceptualized as a bundled social VR musical exergame designed to support autonomy, competence, and relatedness simultaneously.

#### Autonomy

Participants are afforded choice through customization of their avatars and selection from a curated library of music genres and movement patterns.

#### Competence

The exergame requires participants to hit virtual targets in synchrony with musical beats. Real-time visual and auditory feedback, performance scores, and adaptive challenge levels are intended to support mastery and perceived capability [33,34].

#### Relatedness and Social Layer

Participants interact in multiuser virtual rooms for collaborative and competitive team tasks, with real-time voice chat enabled to support communication and social connection. Each session will include a minimum of 10 minutes of structured collaborative or competitive team tasks and at least 5 minutes of open voice chat interaction with coparticipants. The social manipulation therefore comprises multiple elements, including peer communication, collaboration, and structured social comparison. Accordingly, the trial tests the added value of this bundled social layer rather than the isolated effect of any single social feature.

#### Active Control Group: VR Solo Musical Exergame

This group will engage in a VR exergame with the same frequency, duration, exercise intensity, game content, and music design as the experimental group. However, all social features will be disabled. This condition is designed to preserve autonomy and competence support while removing the bundled social layer, and it serves as the primary comparator for testing the unique contribution of social interaction.

#### Waitlist Control Group

Participants in this group will receive a standardized digital handbook on mental health. They will be instructed to maintain their usual activities for the 6-week study period. To address ethical considerations, this group will be offered access to the VR intervention after the trial. Comparisons with this group are intended as secondary estimates of effects relative to minimal guidance rather than as the primary test of the social mechanism.

### Outcomes

#### Primary Outcomes

The primary outcome will be the change in symptom severity for depression and anxiety.

#### Depression Symptoms

Depression symptoms will be assessed using the Beck Depression Inventory-II (BDI-II), a 21-item self-report questionnaire with established reliability and validity [35]. The validated Chinese version of the BDI-II will be used in this study [36].

#### Anxiety Symptoms

Anxiety symptoms will be assessed using the Beck Anxiety Inventory (BAI), a 21-item self-report questionnaire with demonstrated reliability and validity [37]. The validated Chinese version of the BAI will be used [38].

#### Secondary and Process Outcomes

##### Basic Psychological Need Satisfaction

Basic psychological need satisfaction will be assessed using the Basic Psychological Need Satisfaction Scale, a 21-item measure with evidence supporting its 3-factor structure and use in relevant populations [39,40]. The validated Chinese version will be used, which has demonstrated adequate psychometric properties in Chinese young adult samples [40].

##### Loneliness

Loneliness will be assessed using the University of California, Los Angeles Loneliness Scale, a 20-item instrument with documented psychometric support in adolescent and young adult samples [41]. The Chinese version of the scale, which has been validated in Chinese adolescent populations, will be used [41].

##### Sense of Presence

Sense of presence will be assessed using the Igroup Presence Questionnaire, a 14-item scale for measuring presence in virtual environments [42,43]. A Chinese version of the Igroup Presence Questionnaire will be used, adapted following standard translation and back-translation procedures [44].

##### Cardiorespiratory Fitness

Cardiorespiratory fitness will be estimated using the Young Men’s Christian Association 3-minute step test, a practical field-based measure with established validity [45,46]. This outcome is treated as a secondary health-related outcome rather than as a primary mechanistic SDT variable.

##### Music-Movement Synchronization

Music-movement synchronization is an objective behavioral metric captured by the VR software, reflecting the accuracy and timing of participants’ physical movements relative to musical beats. Within the SDT framework, this variable is treated as an exploratory behavioral indicator that may reflect task engagement and perceived competence. It is not a primary or confirmatory outcome; any associations with other variables will be interpreted as hypothesis generating.

All self-report and fitness measures will be collected at T0, T1, and T2. In-VR behavioral data will be captured continuously during the intervention period.

### Randomization and Blinding

A computer-generated random number sequence with variable block sizes will be used to allocate participants in a 1:1:1 ratio. Randomization will be stratified by gender and primary presenting concern (depression vs anxiety) to ensure balanced group allocation on these prognostically important variables. The allocation sequence will be concealed from research staff responsible for recruitment. Because of the nature of the intervention, participants and session facilitators cannot be blinded. Outcome data will be collected primarily through self-report instruments administered through a secure platform, and analyses will be conducted on deidentified group codes until the primary models are finalized.

### Safety Monitoring and Adverse Events

Adverse events will be monitored throughout the trial. Participants will be asked about dizziness, nausea, eyestrain, disorientation, social distress, and symptom worsening at each VR session and at formal assessment points. The Simulator Sickness Questionnaire (SSQ) will be administered at the end of every VR session to track habituation trajectories and detect differential cybersickness across groups; SSQ scores will be examined as a potential covariate in sensitivity analyses. Sessions may be paused or discontinued if participants experience clinically meaningful discomfort. All adverse events and withdrawals will be recorded.

Predefined clinical thresholds will be used to identify participants requiring immediate attention. A BDI-II total score exceeding 28 (indicating severe depression) or any endorsement of suicidal ideation on BDI-II item 9 (score ≥1) at any assessment point will trigger immediate clinical review. Similarly, a BAI total score exceeding 25 (indicating severe anxiety) will prompt review. Self-report assessments will be scored in real time at each formal assessment point, with automatic flagging of scores exceeding these thresholds. Session facilitators will also conduct a brief verbal check-in before and after each VR session to monitor participant well-being.

When a clinical threshold is met, the session facilitator will immediately notify the principal investigator, who will conduct or arrange a clinical risk assessment within 24 hours. Participants meeting clinical concern criteria will be referred to the psychological counseling center at Hunan Traditional Chinese Medical College for nonurgent concerns or to the psychiatric emergency department at the nearest affiliated hospital for acute crises. The principal investigator (GZW) will serve as the designated safety officer responsible for overseeing all safety monitoring procedures and referral decisions. Participants who are referred for clinical care may continue in the trial if deemed safe by the assessing clinician or may be withdrawn if continued participation poses a risk to their well-being.

### Data Collection and Analysis

Data collection will be conducted via a secure online survey platform and directly from the VR software. All primary analyses will follow the intention-to-treat principle and will include all randomized participants with available data.

### Data Management

All data will be entered into a secure electronic database with verification procedures. Identifiable information will be stored separately from outcome data on encrypted institutional servers, accessible only to authorized members of the research team.

For the primary outcomes, linear mixed-effects models will be used to examine group, time, and group×time effects on BDI-II and BAI scores. Separate models will be fitted for depression and anxiety outcomes, with participant-level random intercepts to account for repeated measures over time. Gender and primary presenting concern (the stratification variables) will be included as covariates, and baseline symptom severity will be considered in model specification as appropriate. The primary planned contrasts will compare the experimental group with the active control group at T1 and T2; comparisons involving the waitlist control group will be secondary and should be interpreted with caution, given the expectancy and novelty confounds inherent in waitlist comparisons. Post hoc pairwise comparisons will be adjusted for multiple testing using a Holm procedure. Secondary outcomes will be analyzed using analogous mixed-model approaches. Cybersickness (SSQ scores) will be examined as a potential covariate in sensitivity analyses to assess whether differential simulator sickness across groups confounds the primary results. Missing data assumptions will be examined, with maximum likelihood estimation in mixed models as the primary approach and multiple imputation and per-protocol analyses used as sensitivity analyses where appropriate. Exploratory analyses will examine whether changes in Basic Psychological Need Satisfaction Scale scores are associated with changes in BDI-II and BAI scores and whether music-movement synchronization moderates selected outcomes; these analyses will be interpreted as hypothesis generating rather than confirmatory causal tests.

### Ethical Considerations

This study was approved by the ethics committee of Hunan Traditional Chinese Medical College on September 8, 2025 (YXLL202509006). The trial was prospectively registered at ClinicalTrials.gov on March 15, 2026 (NCT07482852), prior to participant enrollment. Freely given, written informed consent will be obtained from all participants before enrollment. Participation will be voluntary, and participants may withdraw at any time without penalty. Identifiable information will be stored separately from study data on encrypted institutional servers and will be accessible only to authorized members of the research team. Analyses and publications will use deidentified data only, and no personally identifying information will be reported. Any reimbursement or compensation will be provided only as approved by the ethics committee and described in the participant information sheet.

## Results

**Figure 1:**
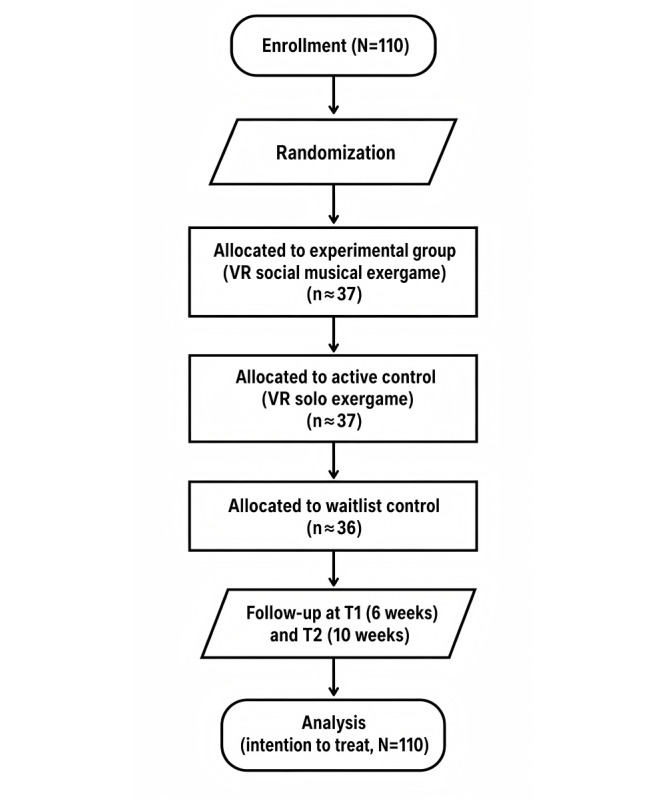
Study flow diagram for the virtual reality (VR)-based social musical exergame.

As of April 2026, ethics approval had been obtained, internal institutional funding had been secured, and the trial had been prospectively registered at ClinicalTrials.gov. The study status was “not yet recruiting” at the time of manuscript revision, no participants had been enrolled, and no outcome data had been collected or analyzed. Recruitment is anticipated to begin in May 2026, with primary completion on March 1, 2028, study completion on May 1, 2028, and publication of the primary findings expected in late 2028. The study flow diagram is presented in Figure 1.

## Discussion

This protocol describes a theoretically informed but pragmatically bundled VR social musical exergame for young adults with mild to moderate depression or anxiety. We hypothesize that participants in the experimental group will show greater improvements in depressive and anxiety symptoms than those in the matched solo VR condition and the waitlist control, with the most policy-relevant efficacy comparison being the experimental group vs the active control group. Comparisons involving the waitlist group are secondary and should be interpreted with caution, as the novelty and expectancy effects associated with VR technology may inflate observed differences relative to an untreated control.

Several features strengthen the design. The trial includes an active comparator matched on VR exposure, music, movement, and dose; repeated assessment across baseline, the postintervention assessment, and follow-up; both subjective and behavioral outcome indicators; stratified randomization by gender and primary presenting concern; a familiarization session to mitigate novelty effects; and a structured clinical safety protocol with predefined thresholds for symptom worsening. Framing the intervention within SDT provides a coherent rationale for why a socially enriched exergame may be more engaging than conventional low-intensity guidance [33,34], although the bundled design does not permit causal isolation of individual SDT mechanisms.

The protocol also has important limitations that should be considered when interpreting future results. First, the 1-month follow-up captures only short-term stability rather than long-term remission; it cannot assess relapse patterns, sustained behavioral change, or long-term clinical trajectories, and future trials should include follow-up periods of at least 3 to 6 months. Second, the social condition is a bundled manipulation that combines voice chat, collaboration, competition, and visible performance comparison; therefore, the trial cannot isolate the independent effect of each social feature, and the study should be understood as a preliminary multicomponent efficacy trial that may inform future dismantling or factorial designs to identify active therapeutic ingredients. Third, the primary outcomes (BDI-II and BAI) are self-report measures administered in an unblinded trial, which increases susceptibility to expectancy, demand characteristics, and placebo effects, particularly given the immersive and novel nature of the VR intervention; future trials should consider incorporating clinician-administered measures (eg, the Hamilton Depression Rating Scale) or objective biomarkers alongside self-report instruments. Fourth, although the intervention is informed by SDT, the bundled design does not isolate or manipulate autonomy, competence, and relatedness independently; any associations between need satisfaction and symptom change are therefore exploratory and cannot establish causal mediation. Fifth, differential cybersickness between the social and solo VR conditions could confound group comparisons, as more complex social interactions may elicit different levels of simulator sickness. Sixth, the study is powered for the primary clinical outcomes but not specifically for mediation or moderation analyses, which are therefore exploratory and hypothesis generating. Finally, although the eligibility range has been narrowed to 18 to 25 years to reduce developmental heterogeneity, individual differences within this developmental period may still influence intervention response.

If the intervention demonstrates favorable short-term effects and acceptable safety, the findings may support a scalable and engaging digital mental health approach that is feasible on consumer-grade VR hardware. The protocol may also inform future dismantling studies that test specific social features, as well as trials with longer follow-up and broader multisite implementation.

The results of this study will be disseminated through peer-reviewed publications and scientific conferences. In addition to reporting clinical outcomes, future publications should clarify feasibility, adherence, adverse events, and the interpretive limits of exploratory process analyses.

## Data Availability

The deidentified dataset generated during this trial will be available from the corresponding author on reasonable request after publication of the primary results, subject to ethics approval and institutional data-sharing requirements.

## Abbreviations

BAI: Beck Anxiety Inventory
BDI-II: Beck Depression Inventory-II
SDT: self-determination theory
SPIRIT: Standard Protocol Items: Recommendations for Interventional Trials
SSQ: Simulator Sickness Questionnaire
VR: virtual reality

## References

[1] Bear HA, Ayala Nunes L, Ramos G, Manchanda T, Fernandes B, Chabursky S, Walper S, Watkins E, Fazel M (2024). The acceptability, engagement, and feasibility of mental health apps for marginalized and underserved young people: systematic review and qualitative study. J Med Internet Res.

[2] Kessler RC, Berglund P, Demler O, Jin R, Merikangas KR, Walters EE (2005). Lifetime prevalence and age-of-onset distributions of DSM-IV disorders in the National Comorbidity Survey Replication. Arch Gen Psychiatry.

[3] Mondin TC, Konradt CE, de Azevedo Cardoso T, de Avila Quevedo L, Jansen K, de Mattos LD, Pinheiro RT, da Silva RA (2013). Anxiety disorders in young people: a population-based study. Braz J Psychiatry.

[4] Copeland WE, Angold A, Shanahan L, Costello EJ (2014). Longitudinal patterns of anxiety from childhood to adulthood: the Great Smoky Mountains Study. J Am Acad Child Adolesc Psychiatry.

[5] Cruwys T, Haslam SA, Dingle GA, Haslam C, Jetten J (2014). Depression and social identity: an integrative review. Pers Soc Psychol Rev.

[6] Thoits PA, Aneshensel CS, Phelan JC, Bierman A (2012). Self, identity, stress, and mental health. Handbook of the Sociology of Mental Health.

[7] Taylor PJ, Gooding P, Wood AM, Tarrier N (2011). The role of defeat and entrapment in depression, anxiety, and suicide. Psychol Bull.

[8] Baumel A, Muench F, Edan S, Kane JM (2019). Objective user engagement with mental health apps: systematic search and panel-based usage analysis. J Med Internet Res.

[9] Elkes J, Cro S, Batchelor R, O'Connor S, Yu LM, Bell L, Harris V, Sin J, Cornelius V (2024). User engagement in clinical trials of digital mental health interventions: a systematic review. BMC Med Res Methodol.

[10] Smith KA, Ward T, Lambe S, Ostinelli EG, Blease C, Gant T, Gold SM, Holmes EA, Paccoud I, Vinnikova A, Klucken J, Uhlhaas PJ, Sanchez CG, Haining K, Böge K, Lahutina S, Tomelleri L, Ryan S, Torous J, Cipriani A (2025). Engagement and attrition in digital mental health: current challenges and potential solutions. NPJ Digit Med.

[11] Soltani P, Figueiredo P, Vilas-Boas JP (2021). Does exergaming drive future physical activity and sport intentions?. J Health Psychol.

[12] Hung L, Wong J, Upreti M, Kan W (2023). Using virtual reality in long-term care to reduce social isolation. Innov Aging.

[13] Ugur F, Sertel M (2020). The effect of virtual reality applications on balance and gait speed in individuals with Alzheimer dementia: a pilot study. Top Geriatr Rehabil.

[14] Liao YY, Chen IH, Hsu WC, Tseng HY, Wang RY (2021). Effect of exergaming versus combined exercise on cognitive function and brain activation in frail older adults: a randomised controlled trial. Ann Phys Rehabil Med.

[15] de BJ Tailored immersion: adaptive virtual reality as a tool for personalized PTSD treatment. Uppsala University.

[16] Jeyakumar V, Sundaram P, Ramapathiran N, Kannan P, Lim CP, Vaidya A, Chen YW, Jain V, Jain LC (2022). Virtual reality-based rehabilitation gaming system. Artificial Intelligence and Machine Learning for Healthcare.

[17] Brescia K (1998). Defining Music Therapy, Second Edition.

[18] Burrai F, Lupi R, Luppi M, Micheluzzi V, Donati G, Lamanna G, Raghavan R (2019). Effects of listening to live singing in patients undergoing hemodialysis: a randomized controlled crossover study. Biol Res Nurs.

[19] Tarr B, Launay J, Dunbar RI (2014). Music and social bonding: "self-other" merging and neurohormonal mechanisms. Front Psychol.

[20] Lai B, Young R, Craig M, Chaviano K, Swanson-Kimani E, Wozow C, Davis D, Rimmer JH (2023). Improving social isolation and loneliness among adolescents with physical disabilities through group-based virtual reality gaming: feasibility pre-post trial study. JMIR Form Res.

[21] Kenyon K, Kinakh V, Harrison J (2023). Social virtual reality helps to reduce feelings of loneliness and social anxiety during the Covid-19 pandemic. Sci Rep.

[22] Kelson JN, Ridout B, Steinbeck K, Campbell AJ (2021). The use of virtual reality for managing psychological distress in adolescents: systematic review. Cyberpsychol Behav Soc Netw.

[23] Li H, Zhang X, Wang H, Yang Z, Liu H, Cao Y, Zhang G (2021). Access to nature via virtual reality: a mini-review. Front Psychol.

[24] Tarr B, Slater M, Cohen E (2018). Synchrony and social connection in immersive virtual reality. Sci Rep.

[25] Tuominen PP, Saarni LA (2024). The use of virtual technologies with music in rehabilitation: a scoping systematic review. Front Virtual Real.

[26] Sun Y, Shaikh O, Won AS (2019). Nonverbal synchrony in virtual reality. PLoS One.

[27] Wei X, Jin X, Fan M (2022). Communication in immersive social virtual reality: a systematic review of 10 years’ studies. Proceedings of the Tenth International Symposium of Chinese CHI.

[28] Rennung M, Göritz AS (2016). Prosocial consequences of interpersonal synchrony: a meta-analysis. Z Psychol.

[29] Arts E, De Castro BO, Luteijn E, Elsendoorn B, Maric M, Vissers CT (2024). Virtual reality training to improve socio‐emotional functioning in adolescents with developmental language disorders: a multiple baseline effectiveness study. Soc Dev.

[30] Freeman D, Reeve S, Robinson A, Ehlers A, Clark D, Spanlang B, Slater M (2017). Virtual reality in the assessment, understanding, and treatment of mental health disorders. Psychol Med.

[31] Carl E, Stein AT, Levihn-Coon A, Pogue JR, Rothbaum B, Emmelkamp P, Asmundson GJ, Carlbring P, Powers MB (2019). Virtual reality exposure therapy for anxiety and related disorders: a meta-analysis of randomized controlled trials. J Anxiety Disord.

[32] Anderson A, Stankovic A, Cowan D, Fellows A, Buckey J Jr (2023). Natural scene virtual reality as a behavioral health countermeasure in isolated, confined, and extreme environments: three isolated, confined, extreme analog case studies. Hum Factors.

[33] Deci EL, Ryan RM, Van Lange PA, Kruglanski AW, Higgins ET (2012). Self-determination theory. Handbook of Theories of Social Psychology.

[34] Ng JY, Ntoumanis N, Thøgersen-Ntoumani C, Deci EL, Ryan RM, Duda JL, Williams GC (2012). Self-determination theory applied to health contexts: a meta-analysis. Perspect Psychol Sci.

[35] Eser MT, Aksu G (2021). Beck Depression Inventory-II: a study for meta-analytical reliability generalization. Pegem J Educ Instruct.

[36] Wang Z, Yuan C, Huang J, Li Z, Chen J, Zhang H, Fang Y, Xiao Z (2011). Reliability and validity of the Chinese version of Beck Depression Inventory-Ⅱ among depressed patients. Chin J Ment Health.

[37] Oh H, Park K, Yoon S, Kim Y, Lee SH, Choi YY, Choi KH (2018). Clinical utility of Beck Anxiety Inventory in clinical and nonclinical Korean samples. Front Psychiatry.

[38] Cheng SK, Wong CW, Wong KC, Chong GS, Wong MT, Chang SS, Wong SY, Chan CK, Wu KO (2002). A study of psychometric properties, normative scores, and factor structure of the Beck Anxiety Inventory--the Chinese version. Chin J Clin Psychol.

[39] Janić O, Lazić M, Ljevaja M (2025). The Basic Psychological Needs Satisfaction and Frustration Scale (BPNSFS) among Serbian adolescents: testing factor structure and gender measurement invariance. Curr Psychol.

[40] Gu J, Wang JL (2023). Basic psychological needs satisfaction profiles and well-being among Chinese adolescents and Chinese university students: the role of growth mindset. Curr Psychol.

[41] Ip H, Suen YN, Hui LM, Cheung C, Wong SM, Chen EY (2024). Psychometric properties of the variants of the Chinese UCLA Loneliness Scales and their associations with mental health in adolescents. Sci Rep.

[42] Vasconcelos-Raposo J, Bessa M, Melo M, Barbosa L, Rodrigues R, Teixeira CM, Cabral L, Sousa AA (2016). Adaptation and validation of the Igroup Presence Questionnaire (IPQ) in a Portuguese sample. Presence.

[43] Panahi-Shahri M, Fathi-Ashtiani A, Azad-Fallah P, Montazer G (2009). Reliability and validity of Igroup Presence Questionnaire (IPQ). J Behav Sci.

[44] Schubert T, Friedmann F, Regenbrecht H (2001). The experience of presence: factor analytic insights. Presence.

[45] Kieu NT, Jung SJ, Shin SW, Jung HW, Jung ES, Won YH, Kim YG, Chae SW (2020). The validity of the YMCA 3-minute step test for estimating maximal oxygen uptake in healthy Korean and Vietnamese adults. J Lifestyle Med.

[46] Weisstaub G, González J, Orizola I, Borquez J, Monsalves-Alvarez M, Lera L, Troncoso R, Sepúlveda C (2025). Validity and reliability of the step test to estimate maximal oxygen consumption in pediatric population. Sci Rep.

